# Integrating clinical and genomic landscape analysis of perineural invasion identify ACTA1 as an oncogene for oral squamous cell carcinoma

**DOI:** 10.3389/fcell.2024.1458879

**Published:** 2024-10-15

**Authors:** Sheng Chen, Tongchao Zhao, Yuxian Song, Xiaofeng Huang, Yanhong Ni, Liang Ding, Yong Fu, Qingang Hu, Yi Wang

**Affiliations:** ^1^ Central Laboratory, Nanjing Stomatological Hospital, Medical School of Nanjing University, Nanjing, Jiangsu, China; ^2^ Department of Oral Pathology, Nanjing Stomatological Hospital, Medical School of Nanjing University, Nanjing, Jiangsu, China; ^3^ Department of Stomatology, Huashan Hospital, Fudan University, Shanghai, China; ^4^ Department of Oral Pathology Ninth People’s Hospital, College of Stomatology Shanghai Jiao Tong University School of Medicine, Shanghai, China

**Keywords:** PNI, oral squamous cell carcinoma, TCGA, ACTA1, immunothearpy

## Abstract

**Background:**

Perineural invasion (PNI) has been shown to be a key pathological feature of several types of cancer, including oral squamous epithelial carcinoma (OSCC). However, the overall clinical and genomic landscape of PNI^+^ OSCC are still unclear, and the molecular mechanism of PNI remains to be further investigated.

**Methods:**

279 OSCC samples were extracted from the TCGA database and grouped according to PNI. The clinicopathological information, prognostic and survival analyses were performed. The Cibersort algorithm and ESTIMATE algorithm was used to estimate the impacts on proportion of immune cells, immune score and stromal score by PNI. Immunotherapy prediction analysis was also performed. 167 differentially expressed genes were screened for functional enrichment analysis. Actin α1 (ACTA1) protein, which was significantly upregulated in the PNI^+^ group, was selected for validation in our OSCC patient’s cohort (n = 70). We next analyzed the ratio and absolute number of key immunocytes in peripheral blood of OSCC patients according to Actin α1 expression by flow cytometry.

**Results:**

PNI was more likely to occur in patients with advanced tumors and worse prognosis. Immunomodulation analyses showed that T cells follicular helper and cells were significantly lower, but M2 macrophages and total stromal score was significantly higher in PNI^+^ OSCC. Immunotherapy prediction analyses showed that PNI^+^ OSCC may be more sensitive to CTLA4 inhibitor treatment. 167 differentially expressed genes were identified and enriched in muscle structure and cell movement-related pathway. Among them, Actin α1 (ACTA1) was significantly upregulated in PNI^+^ advanced OSCC with worse clinical outcome whose had relatively low ratio of CD3^+^CD8^+^ circulating cytotoxic T cells.

**Conclusion:**

PNI^+^ OSCC patients with upregulated of Actin α1 could benefit from cytotoxic T cell-mediated immunotherapy.

## Introduction

Oral squamous cell carcinoma (OSCC) is one of the most common malignant tumors in the head and neck region (Bray et al., 2018), which has the characteristics of relatively insidious site of onset, mostly in the middle and late stages when it is detected and diagnosed, and has the characteristics of strong aggressiveness, easy to metastasize, and poor prognosis, etc. The five-year survival rate of patients with squamous oral cancer is still maintained at about 60% after treatment (Fan et al., 2022). As the tumor progresses, patients with squamous oral cancer often die from tumor recurrence and metastasis.

In recent years, with increased attention to the role of nerves in the tumor microenvironment, scholars have found that perineural invasion (PNI), a process of tumor invasion of nerves, is an important pathological feature of many malignancies, including tumors of the head and neck, pancreas, colorectum, prostate, biliary tract, and stomach (Schmitd et al., 2022; Yang et al., 2020; Yin et al., 2021; Nagakawa et al., 1993; Li et al., 2023; Albergotti et al., 2017). Multiple studies have shown that PNI is associated with morbidity, suggesting a worse prognosis. In a series of 381 patients who received for low-risk prostate cancer, Beard et al. reported that the 5-year prostate-specific antigen (PSA) failure-free survival rate was 50% versus 80% in patients with and without PNI in their needle biopsy specimens, respectively (Beard et al., 2004). Positive PNI status in pancreatic cancer predicts decreased survival, often independent of stage, but treatment remains unchanged by PNI status (Jurcak and Zheng, 2019). All above reports suggest that PNI has important clinical significance, but the mechanism of its occurrence is still unclear.

In this paper, we studied the differences between PNI-positive and PNI-negative oral squamous carcinomas based on TCGA data, respectively, in terms of prognosis, gene expression, tumor microenvironment, immune scores, stromal scores, prediction of tumor drug resistance, and prediction of immunotherapeutic efficacy, etc. We selected the relevant obvious differentially expressed genes for validation, with the aim of providing clues for further research.

Additionally, we found that the gene encoding the ACTA1 protein was significantly upregulated in PNI-positive samples. ACTA1 encode actin α1, one of the key structural proteins that make up the cytoskeleton, and plays a role in functions such as cell division, migration, and vesicular transport (Ravenscroft et al., 2011). In pancreatic ductal adenocarcinoma, ACTA1 expression is increased in stromal progenitor cells and fibroblast-like cells during carcinogenesis (Peng et al., 2019). In basal-like breast cancer, ACTA1 is a biomarker associated with chemotherapy resistance (Jaaks et al., 2022). So, we hypothesized that after tumor cells invaded nerve tissues, the invaded nerves could upregulate ACTA1 expression in tumor cells, leading to epithelial-mesenchymal transition of the cells, which promoted the invasion and metastasis of tumor cells.

## Materials and methods

### Data and sample collection

To conduct our analysis, we downloaded the expression data, clinical data and phenotypic data of HNSC from the TCGA database (expression data: https://tcga.xenahubs.net/download/TCGA.HNSC.sampleMap/HiSeqV2.gz, clinical data: https://tcga.xenahubs.net/download/survival/HNSC_survival.txt.gz, phenotype matrix: https://tcga.xenahubs.net/download/TCGA.HNSC.sampleMap/HNSC_clinicalMatrix). Sample TMB data from https://gdc.cancer.gov/about-data/publications/PanCan-CellOfOrigin. Sample mRNAsi score data were obtained from PMID: 29625051 Attachment 1. From the above data, we finally filtered 279 OSCC samples for subsequent analysis (Table 1). R programming (vision 3.6.3) was applied for integrating, analyzing, and visualizing the data. Further integration of sample PNI, Age, Gender, pathologic_T, pathologic_stage information, and visualization of grouping results using the R package circlize (v0.4.10).

**TABLE 1:** 279 OSCC samples collected from the TCGA database.

Features	n (%)
Gender	
Male	187 (67.0)
Female	92 (33.0)
Age (years): Mean (Median)	62 (61)
Pathologic_T	
T1	25 (9.0)
T2	92 (33.0)
T3	55 (19.7)
T4	107 (38.4)
Pathologic_N	
N0	133 (47.7)
N1	47 (16.8)
N2	97 (34.8)
N3	2 (0.7)
Pathologic TNM	
Stage I	16 (5.7)
Stage II	52 (18.6)
Stage III	54 (19.4)
Stage IV	157 (56.3)
Tumor site	
Alveolar Ridge	17 (6.1)
Buccal Mucosa	21 (7.5)
Floor of mouth	56 (20.1)
Hard Palate	5 (1.8)
Oral Tongue	112 (40.1)
Oral Cavity	68 (24.4)
PNI	
Yes	153 (54.8)
No	126 (45.2)

PNI, perineural invasion.

The resected tissue samples were collected from 70 primary OSCC patients who underwent resection surgery from January 2016 to June 2016 (Table 2). All methods used for this study were approved by the Ethics Committee of Nanjing Stomatology Hospital (NJSH-2023NL-03). The study was carried out in accordance with the Declaration of Helsinki. Written informed consent was obtained from all the patients.

**TABLE 2:** 70 primary OSCC patients who underwent resection surgery in Nanjing Stomatology Hospital.

		Total N(%)		ACTA1 in TCs			
				Low		High	
All cases		70	100.00%	34	48.57%	36	51.43%
Gender	Female	26	37.14%	11	32.35%	15	41.67%
	Male	44	62.86%	23	67.65%	21	58.33%
Age	≤60	31	44.29%	14	41.18%	17	47.22%
	>60	39	55.71%	20	58.82%	19	52.78%
T stage	Ⅰ-Ⅱ	37	52.86%	22	64.71%	15	41.67%
	Ⅲ-Ⅳ	33	47.14%	12	35.29%	21	58.33%
LNM	No	36	51.43%	20	58.82%	16	44.44%
	Yes	34	48.57%	14	41.18%	20	55.56%
PNI	No	43	61.43%	26	76.47%	19	52.78%
	Yes	27	38.57%	8	23.53%	17	47.22%

PNI, perineural invasion; LNM, lymph node metastasis; TCs, tumor cells.

### Analysis of differences in mRNAsi scores

The mRNAsi score information of the samples was downloaded from PMID:29625051 Attachment 1, and then based on PNI, box line plots were plotted using the R package ggpubr, and the significance of the difference was tested using t. test.

### Immune infiltration analysis

The CIBERSORT algorithm can infer the proportion of 22 immune cells within a sample from the expression of some genes. Therefore, we first extracted the expression data of the feature genes from the complete expression data to obtain the expression matrix of the feature genes, and then used the R package CIBERSORT (v1.03) to calculate the proportion of immune cells in different groups of samples by combining with the existing immune cell signature files.

### ESTIMATE assessed the difference in immunity scores, matrix scores

The R package ESTIMATE (v1.0.13) was used to calculate the immunization score, matrix score of the two groups of samples, then the R package ggpubr (v0.4.0) was used to plot the box line graph and t. test was used to test the significance of the difference.

### Immunotherapy prediction

Previous studies have found that in chronic infectious diseases, combined blockade of PD-1 (Yi et al., 2022) and CTLA-4 can partially restore the biological function of CD8^+^ T cells (Rowshanravan et al., 2018), so the use of PD-1 and CTLA-4 inhibitors may have a better effect on the treatment of tumors, but clinical studies have found that the use of the relevant inhibitors can only alleviate the disease in some patients.

SubMap, a GenePattern online analysis module, can utilize gene expression information to merge two datasets with different traits by functional enrichment mapping, which not only eliminates batch effects, but also predicts the probability of unaccounted traits in the original dataset.

In order to predict whether the relevant immunosuppressant has a therapeutic effect on the high- and low-risk groups, SubMap is used to map the PNI grouped samples to the melanoma samples with the inhibitor treatment information, which in turn predicts the possible effect of the PNI grouping of OSCC on the two inhibitor treatments.

### Drug resistance prediction

The R package pRRophetic (v0.5) was utilized to predict the sensitivity of different subtypes to specific drugs. 10 drugs (BIBW2992, Cisplatin, Docetaxel, Erlotinib, Etoposide, Gefitinib, Gemcitabine, Paclitaxel, Paclitaxel, Vinorelbine, PF_02341066) were used to calculate the half maximal inhibitory concentration (IC50) of different subgroups of samples, and then the five classes with significant differences in sensitivities were selected to plot box plots.

### Differential gene expression analysis

The R package limma (v3.42.2) was utilized for the acquisition of differentially expressed genes with a correlation threshold of |logFC|>1 & FDR <0.05, and then the R packages ggplot2 (v3.3.2) and gplots (v3.1 0) were used for the visualization of differentially expressed genes. Further the differentially expressed genes were subjected to functional enrichment analysis, in which clusterProfiler (v3.14.3) was utilized for enrichment analysis, org. Hs.e.g.,.db (v3.10.0) was used to convert the gene names, and GOplot (v1.0.2) and ggplot2 were used for visualization. Finally, the R package GEOquery (v2.54.1) was utilized to download the GEO data GSE9792, to verify whether the top 10 genes differentially expressed in the TCGA data were similarly differentially expressed, and the R package ggpubr was used to draw box line plots, and to test the significance of the differences with t. test.

### Immunohistochemistry and quantification

The protocol of IHC of formalin-fixed paraffin-embedded sections and scoring details of IHC was performed as previously described. Specifically, IHC was performed on 3-µm formalin-fixed paraffin-embedded sections using anti-Actin (1:200 dilution; ab1801; Abcam). Slides were deparaffinized with xylene and rehydrated in an ethanol series. Antigen retrieval was performed with 10 mmol/L citric acid (pH 6.0) in a pressure cooker. Then, endogenous peroxidase activity was blocked with a 3% hydrogen peroxide solution. After washing in phosphate buffered saline (PBS; pH 7.4) three times, slides were incubated with primary antibodies against actin (ab1801; Abcam) at 4°C overnight. After washing in PBS three times, Polink-2 plus HRP Detection Kit was used as the secondary antibody at 37°C for 40 min. Finally, slides were developed in diaminobenzidine (DAB). And nucleus stained with hematoxylin. The IHC staining results of Actin α1 were independently and double blindly evaluated by two senior pathologists who did not know the patients’ data, and the average values were calculated for further analysis. IHC staining was scored according to the percentage of positive cells and staining intensity. The percentage of stained cells was defined as 0 = 0–5%; 1 = 6%–25%; 2 = 26%–50%; 3 = 51%–75%; and 4 = 75–100%. Staining intensity was defined as follows: 0 = negative staining; 1 = weak staining; 2 = moderate staining; and 3 = strong staining. The IHC score was calculated by multiplying the grade of the staining intensity by that of the staining percentage. High and low expressions of Actin α1 were defined by the median of IHC scores.

### Flow cytometry assay

For the cell subtypes of PBMC analysis, cells were collected and washed with PBS twice and then suspended in 200 μL PBS. For enumeration of mature human T (CD3^+^) cells, helper/inducer T (CD3^+^ CD4^+^) cells, cytotoxic T (CD3^+^ CD8^+^) cells, B (CD19^+^) cells, and NK(CD3^−^ CD16^+^/orCD56^+^) lymphocytes, CD3-FITC/CD8-PE/CD45-PerCP/CD4-APC reagent, BD Multitest CD3-FITC/CD16-PE CD56-PE/CD45-PerCP/CD19-APC reagent were used according to the manufacturer’s instructions, respectively (Cat No.340503, BD Multitest™), then quantified by flow cytometry on a FACS Calibur instrument.

### Statistical analysis

SPSS 18.0 and GraphPad Prism 8.0 software packages were used for data analysis and graphic processing. Visualization and statistical analysis were performed using R (v3.6.3) and prism graphpad (RRID: SCR_000306, https://scicrunch.org/resolver/SCR_000306). All distribution data are expressed as mean ± standard deviation. Differences between the PNI+ and PNI- groups were analyzed using t. test. *p* < 0.05 was considered statistically significant.

## Results

### Pathologic characterization of PNI^+^ OSCC and prognosis analysis

**FIGURE 1:**
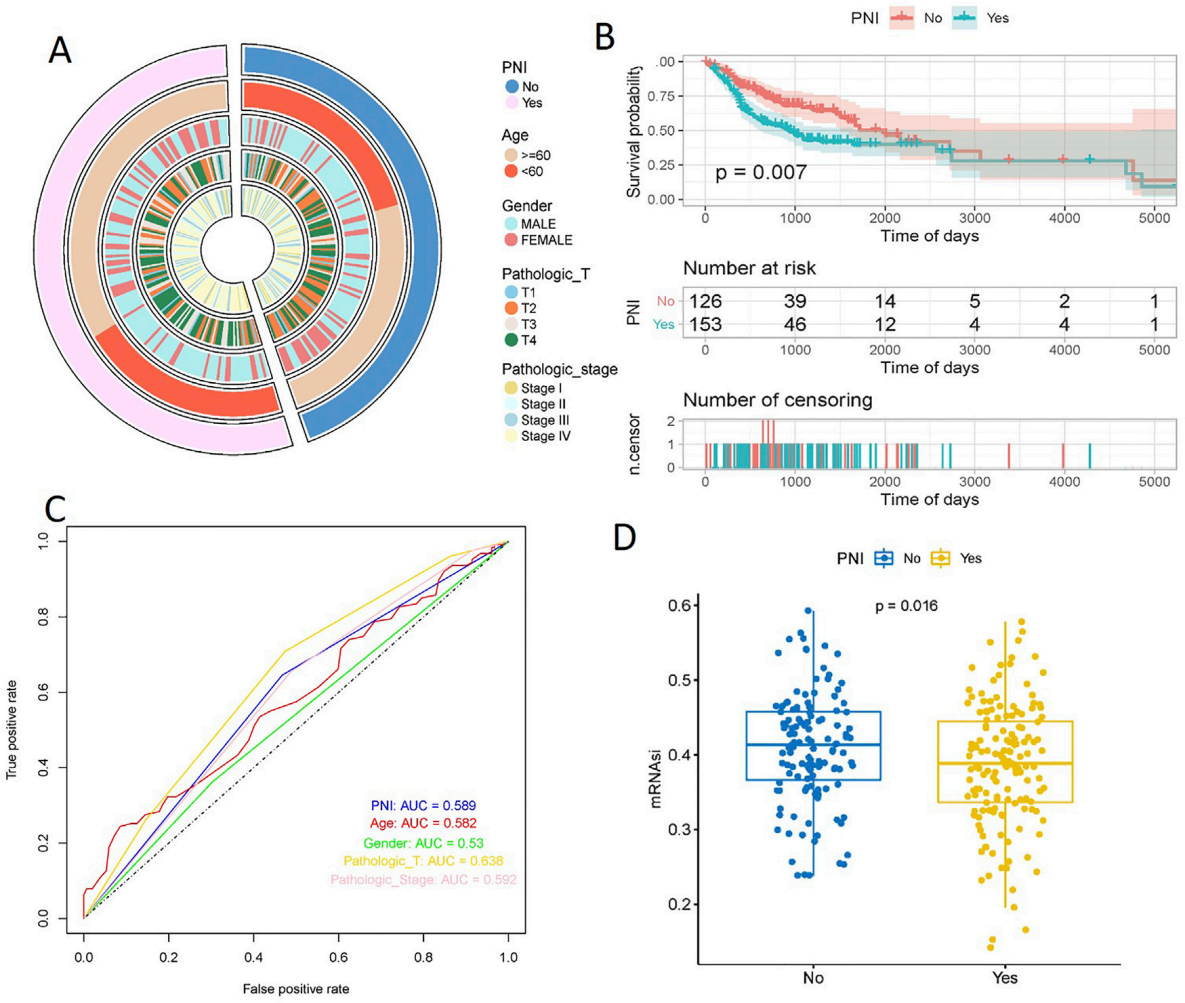
Pathologic characterization of PNI^+^ OSCC and prognosis analysis. t. test showed significant differences in the distribution of Pathologic_T (*p =* 0.041) and Pathologic_stage (*p =* 0.0069) **(A)**. KM curves showed a significant difference in survival between the two groups of samples (*p =* 0.007), with a significantly slower decline in the survival curves of the PNI^+^ samples **(B)**. ROC curves showed that Pathologic_T was the best predictor (*AUC =* 0.638), followed by PNI (*AUC =* 0.589), Pathologic_stage (*AUC =* 0.592), and Age (*AUC =* 0.582); Gender was the worst predictor (*AUC =* 0.53) **(C)**. The mRNAsi scores were significantly higher in PNI^−^ samples than in PNI^+^ samples (*p =* 0.016) **(D)**.

Based on PNI, 279 samples were divided into two groups of PNI-positive and PNI-negative, and the information of Age, Gender, Pathologic_T, Pathologic_stage of the samples were extracted (Table 1), and the distribution of the above information in the PNI grouping was statistically analyzed, and the results showed that more PNI^+^ samples of more Pathologic_T were mostly T3 and T4. Pathologic_stage was mostly Stage III and Stage IV. Further t. test was used to detect the significance of the distribution of nerve infiltration samples, and the results showed that the difference in the distribution of Pathologic_T (*p =* 0.041) and Pathologic_stage (*p =* 0.0069) of PNI + samples was significant (Figure 1A).

Plotting the Kaplan-Meier curves based on the PNI groupings showed a significant difference in survival between the two groups (*p =* 0.007), with the survival curves of the PNI^−^ samples declining significantly slower than those of the PNI^+^ samples (Figure 1B). In order to explore the effect of PNI in prognosis, PNI, Age, Gender, Pathologic_T, and Pathologic_stage were analyzed using cox one-way regression, respectively, and ROC curves were plotted, and the results showed that the predictive effect of Pathologic_T was the best (AUC = 0.638), and that of PNI (AUC = 0.589), PNI (AUC = 0.589), Pathologic_stage (AUC = 0.592), and Age (AUC = 0.582) were the next best predictors; and Gender was the worst predictor (AUC = 0.53). Taken together, this suggests that using PNI as a single prognostic factor is a poor predictor, and it is possible that combining the effects of the factors may lead to better prognostic results (Figure 1C).

Stemness indices are indicators describing the similarity between tumor cells and stem cells. mRNAsi score is an index calculated based on the expression data, and the index ranges from 0 to 1, which is approximately close to 1 indicating that the lower the degree of cell differentiation, the stronger the characteristics of stem cells, analyzing the difference in the mRNAsi scores of the two groups, it was found that mRNAsi scores of the nerve non-infiltrated samples were significantly higher than those of the nerve-infiltrated samples was significantly higher than that of PNI^+^ samples (*p =* 0.016) (Figure 1D).

### The impacts of PNI on the stromal OSCC microenvironment

**FIGURE 2:**
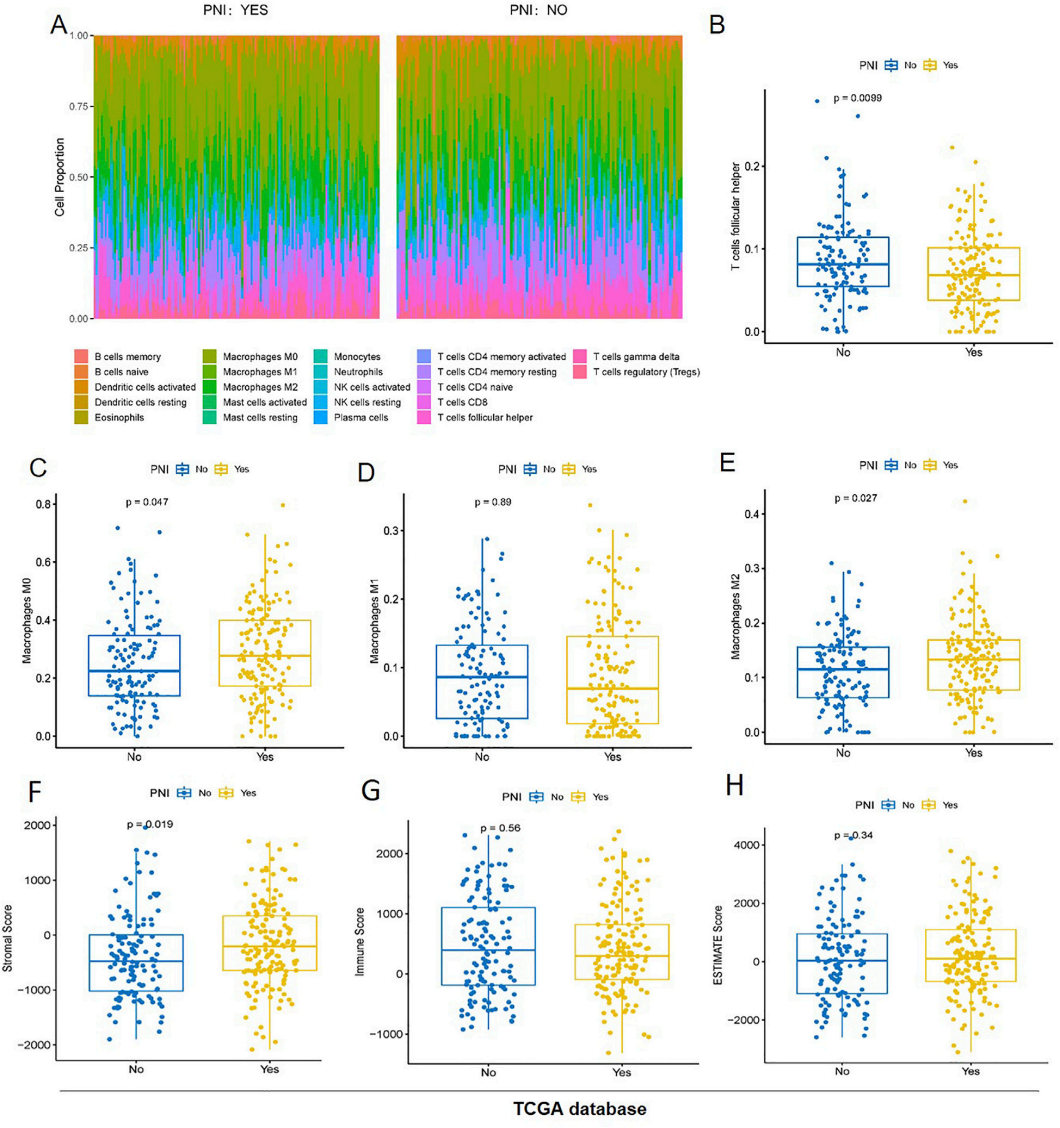
The proportion of immune cells in the two groups of samples **(A)** T cells follicular helper was significantly higher in the PNI^−^ samples than in the PNI^+^ samples **(B)**. Only macrophages, especially the M2 tumor-associated macrophages was significantly higher in the PNI^+^ OSCC **(C–E)**. The stroma score was significantly different (*p =* 0.019) **(F)**. The immunity score **(G)** and the ESTIMATE score **(H)** have no significant difference.

The Cibersort algorithm was used to calculate the proportion of immune cells in the two groups of samples (Figure2A), and the results showed that only the proportion of T cells follicular helper was significantly higher in the PNI^−^ samples than in the PNI^+^ samples (Figure2B), and only macrophages, especially the M2 tumor-associated macrophages was significantly higher in the PNI^+^ OSCC (Figures 2C–E). The ESTIMATE algorithm was used to assess the total immune score and stromal score of the two groups of samples, and the box line plot results showed that the difference between the two groups of samples was significant only in the stromal score (*p =* 0.019) (Figure2F), and the difference between the two groups of samples in the immune score was not significant (Figure2G). The difference between the two groups of samples in ESTIMATE Score was also not significant (Figure2H).

### Immunotherapy response prediction of PNI^+^ OSCC patients

**FIGURE 3:**
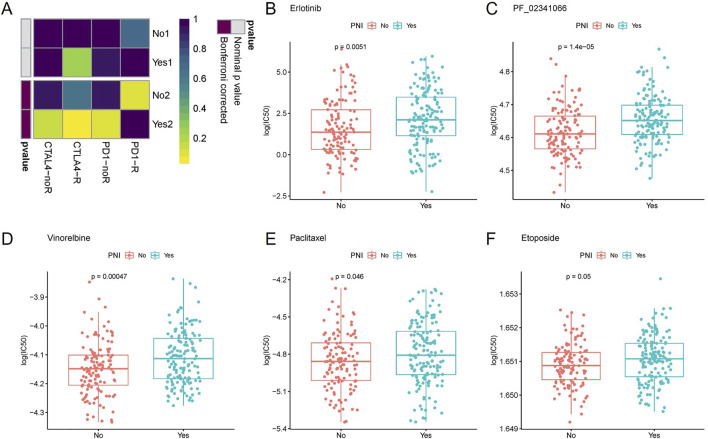
Inferring from the gene expression of each group, the nerve-infiltrated samples were more sensitive to CTLA4 inhibitor (corrected *p =* 0.030), whereas the sensitivity of both groups to PD-1 inhibitor did not reach a significant level **(A)**. The sensitivity of PNI^+^ samples to Erlotinib **(B)**, PF_02341066 **(C)**, Vinorelbine **(D)**, Paclitaxel **(E)** and Etoposide **(F)** varied more significantly.

Based on GenePattern’s online analysis module SubMap, the dataset mapping of OSCC samples to inhibitor-treated melanoma samples initially predicted immunotherapy differences in PNI subgroups. Extrapolating from the gene expression profiles of the groups, the nerve-infiltrated samples were more sensitive to the CTLA4 inhibitor (corrected *p =* 0.030), implying better efficacy for nerve-infiltrated patients, while neither group reached a significant level of sensitivity to the PD-1 inhibitor, implying that the inhibitor was less efficacious for both subgroups (Figure 3A).

Further drug sensitivity prediction for different PNI subgroup samples and statistical analysis of variance revealed (t-test) that the sensitivity of PNI subgroup samples to Vinorelbine, Paclitaxel, PF_02341066, Etoposide, Erlotinib. The sensitivity of PNI^+^ samples to the above drugs was higher than that of PNI^−^ samples. The findings may provide clues for the use of these drugs in the treatment of different subtypes of patients (Figures 3B–F).

### Differential expression gene analysis and validation in PNI^+^ OSCC

**FIGURE 4:**
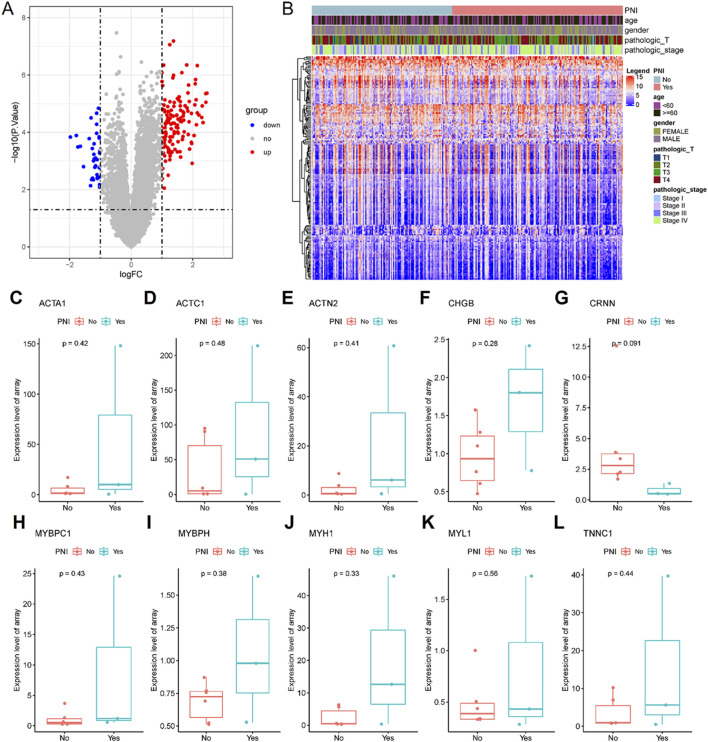
167 differentially expressed genes were screened, of which 131 were upregulated and 36 were downregulated. **(A)** Differential gene volcano plot; **(B)** Differential gene expression heatmap. The all top 10 differentially expressed genes (some genes were not expressed in the GSE9792 data and were directly deferred) were expressed in the GSE9792 samples in the same way as in the TCGA samples **(C–L)**.

The extraction of differentially expressed genes in nerve-infiltrated samples relative to PNI^−^ samples (Yes-No) was performed using the R package limma. The expression matrix of 20,091 genes was obtained by removing low-expression genes with the threshold of the sum of gene expression in all samples being greater than 2. Further, 167 differentially expressed genes were screened with the threshold of |logFC|>1 & FDR<0.05, of which 131 were upregulated and 36 were downregulated (Figures 4A, B; Table 3). Among these genes, DES, ACTC1, MYBPH, MYH1, ACTA1, MYL1, MYL2, ACTN2, CHGB and CSRP3 were the top 10 significantly highly expressed genes.

**TABLE 3:** Differential expression gene in PNI + OSCC.

Gene	logFC	Gene	logFC
DES	2.46176760	MYOZ1	1.25780980
ACTC1	2.42905850	POPDC3	1.25512210
MYBPH	2.42228950	DDIT4L	1.25278020
MYH1	2.33612140	TTN	1.25247440
ACTA1	2.30865180	LDB3	1.22708940
MYL1	2.29442460	CA3	1.21724620
MYL2	2.19937110	KANK4	1.21680410
ACTN2	2.14944030	ADCY2	1.21385170
CHGB	2.14887570	MMP13	1.20814510
CSRP3	2.12074490	CACNG6	1.20606550
MYLPF	2.11051560	CST1	1.20411130
ANKRD1	2.09241060	RAPSN	1.20101390
MYOG	2.08200060	TRIM55	1.19585500
KBTBD10	2.06873330	TMOD4	1.19130200
MYBPC1	2.04339540	MURC	1.19080550
MYH2	1.98443840	PRUNE2	1.16659510
NRAP	1.96822120	SCN5A	1.16136060
TNNC1	1.95905950	MMP7	1.13442860
MYH7	1.95593950	MMP10	1.12628480
XIRP2	1.89978480	PPP1R3A	1.12269030
SMPX	1.89017090	STAC3	1.11996060
TCAP	1.88160280	S100A1	1.11986480
CASQ2	1.86686600	SGCG	1.11791340
MYBPC2	1.85552270	FREM2	1.11547800
HSPB7	1.84519860	SYPL2	1.11227800
ASB5	1.84264390	SOHLH2	1.09995900
MYOT	1.81568580	A2BP1	1.09639110
CHRNA1	1.80529160	MMP11	1.09525620
SMYD1	1.75502250	CDH15	1.09502960
TRDN	1.72334840	TFPI2	1.08771480
TNNC2	1.70879460	SGCD	1.08007370
MYH8	1.70783750	ANKRD23	1.07779720
MYF6	1.69116780	AMTN	1.07649580
SLN	1.68387520	APOD	1.06839130
MYPN	1.68365160	NCAM1	1.06177870
LMOD2	1.66383600	PLCB4	1.05559680
MYH3	1.64800970	MYOM1	1.05134520
COX6A2	1.63591700	CILP	1.04667060
CKM	1.60782260	VGLL2	1.02875040
ARPP21	1.60693300	IP6K3	1.02747380
LMOD3	1.60675780	DACT1	1.02547620
CACNG1	1.59784440	SFRP2	1.02487990
DYSFIP1	1.59559240	CASQ1	1.01425600
UNC45B	1.59505660	GRP	1.00807380
TNNT3	1.58750380	COL11A1	1.00537250
HRC	1.55890310	ST8SIA2	1.00426690
CAV3	1.54218540	TMEM8C	1.00384260
MYOZ2	1.52880820	PCP4L1	−1.01490290
CACNA1S	1.52373940	PRSS3	−1.01759670
CHRND	1.52296450	MUC15	−1.02388680
DUSP27	1.51300580	KLK14	−1.02390620
ATP1A2	1.51077920	MUC4	−1.02515900
MB	1.50517680	ALOX12B	−1.03316610
TNNI1	1.50433230	PRSS27	−1.03461560
KBTBD5	1.50152080	TMPRSS11D	−1.03741610
ITGB1BP3	1.48327990	CYP4F12	−1.03825490
HFE2	1.47257350	ASPG	−1.05937200
APOBEC2	1.46773220	MAL	−1.09362840
NEB	1.42482350	TMPRSS11B	−1.09697390
HHATL	1.40680160	?|729,884	−1.10180640
CKMT2	1.40188070	KLK12	−1.10754690
CHRNG	1.40168080	SLC26A9	−1.11824590
TRIM63	1.39829640	NCCRP1	−1.11889560
SGCA	1.38881830	C9orf169	−1.11906550
RBM24	1.37728700	RHCG	−1.15138360
CORO6	1.37450830	IL1F6	−1.15384190
MYL3	1.36571830	OTOP3	−1.17510900
MYL4	1.35791220	KLK13	−1.19795600
PDLIM3	1.35667030	PLA2G4E	−1.20152710
DUSP26	1.35486170	CEACAM5	−1.20826280
THBS4	1.34877080	CEACAM7	−1.21820570
C10orf71	1.34123020	SPRR2F	−1.22098640
TRIM54	1.32858700	SPINK7	−1.25042410
PRKAG3	1.30418650	KRT78	−1.25943010
MUSTN1	1.30387810	KRT24	−1.28130760
TNNI2	1.30205960	CLCA4	−1.29903590
ATP1B4	1.29590530	KRT4	−1.31470290
ENO3	1.28688460	TMPRSS11A	−1.35758980
MYO18B	1.28137570	TGM3	−1.54449780
SCN4A	1.27437990	MUC21	−1.64642280
MYH6	1.26499640	KRT13	−1.72682060
BEST3	1.26476470	SPRR3	−1.77873210
COL10A1	1.26227910	CRNN	−1.97168570
XIRP1	1.26207650		

To validate the differential expression, GSE9792 microarray data containing 9 samples were downloaded from the GEO database, of which 6 were PNI^+^ samples and 3 PNI^−^ samples, and select the top 10 differentially expressed genes (ACAT1, ACTC1, ACTN2, CHGB, CRNN, MYBPC1, MYBPH, MYH1, MYL1 and TNNC1 in the GSE9792, some genes were not expressed in the GSE9792 data, and were directly deferred) that were pulled out of the TCGA database, and the 10 genes grouped samples were all consistent with those in TCGA grouped samples, but the *P*-value was not significant due to the small sample size of GSE9792 (Figures 4C–L).

### PNI^+^ OSCC enrich muscle structure and cell movement-related pathway

**FIGURE 5:**
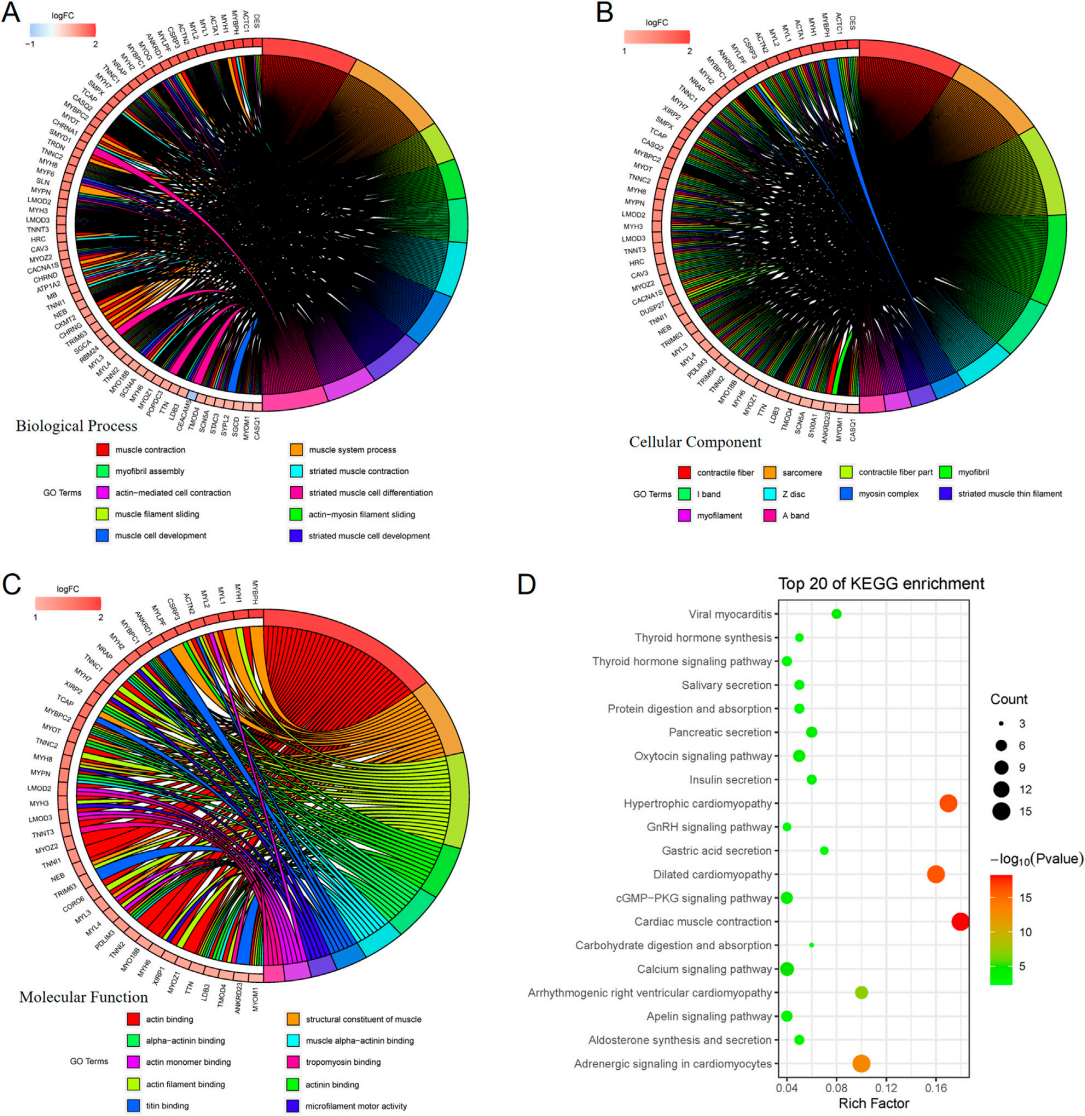
GO enrichment analysis is further divided into 3 parts, namely: Biological Process (BP), Cellular Component (CC), and Molecular Function (MF). BP-enriched pathways are mainly muscle contraction, myofibril assembly, and muscle cell development **(A)**. CC-enriched pathways are mainly contractile fibers, myonodes, myogenic fibers **(B)**. MF-enriched pathways are mainly actin-binding and actin-filament binding **(C)**. KEGG-enriched pathways mainly involve myocardial contraction and secretion of various glands **(D)**.

Functional enrichment analysis of differentially expressed genes was further performed, and GO enrichment analysis was further divided into three parts, i.e., Biological Process (BP) (Figure 5A), Cellular Component (CC) (Figure 5B), and Molecular Function (MF) (Figure 5C), in which the pathways enriched by BP were mainly muscle contraction, myofibril assembly, muscle cell development, etc., the pathways enriched by CC were mainly contractible fibers, myonectin, myogenic fibers, etc., and the pathways enriched by MF were mainly actin, myonodule, myofibril, etc. The pathways enriched by BP are mainly muscle contraction, myofibril assembly, muscle cell development, etc. The pathways enriched by CC are mainly contractile fibers, myonodes, myofibrils, etc. The pathways enriched by MF are mainly actin-binding, actin-filament binding, etc., and the pathways enriched by KEGG are mainly contraction of the myocardium, secretion of various types of glands, etc. The pathways enriched by KEGG are mainly contraction of cardiac muscle, secretion of various types of glands, etc. This suggests that nerve infiltration may be closely related to muscle structure and movement (Figure 5D).

### OSCC patients with high actin α1 expression have poor clinical outcome and low cytotoxic T cells

The actin α1 (ACTA1) protein was selected among the 167 differentially expressed genes that were retrieved from the TCGA database for validation, and ACTA1 is firstly found in skeletal muscle and is highly conserved proteins that play a role in cell motility, structure and integrity.

**FIGURE 6:**
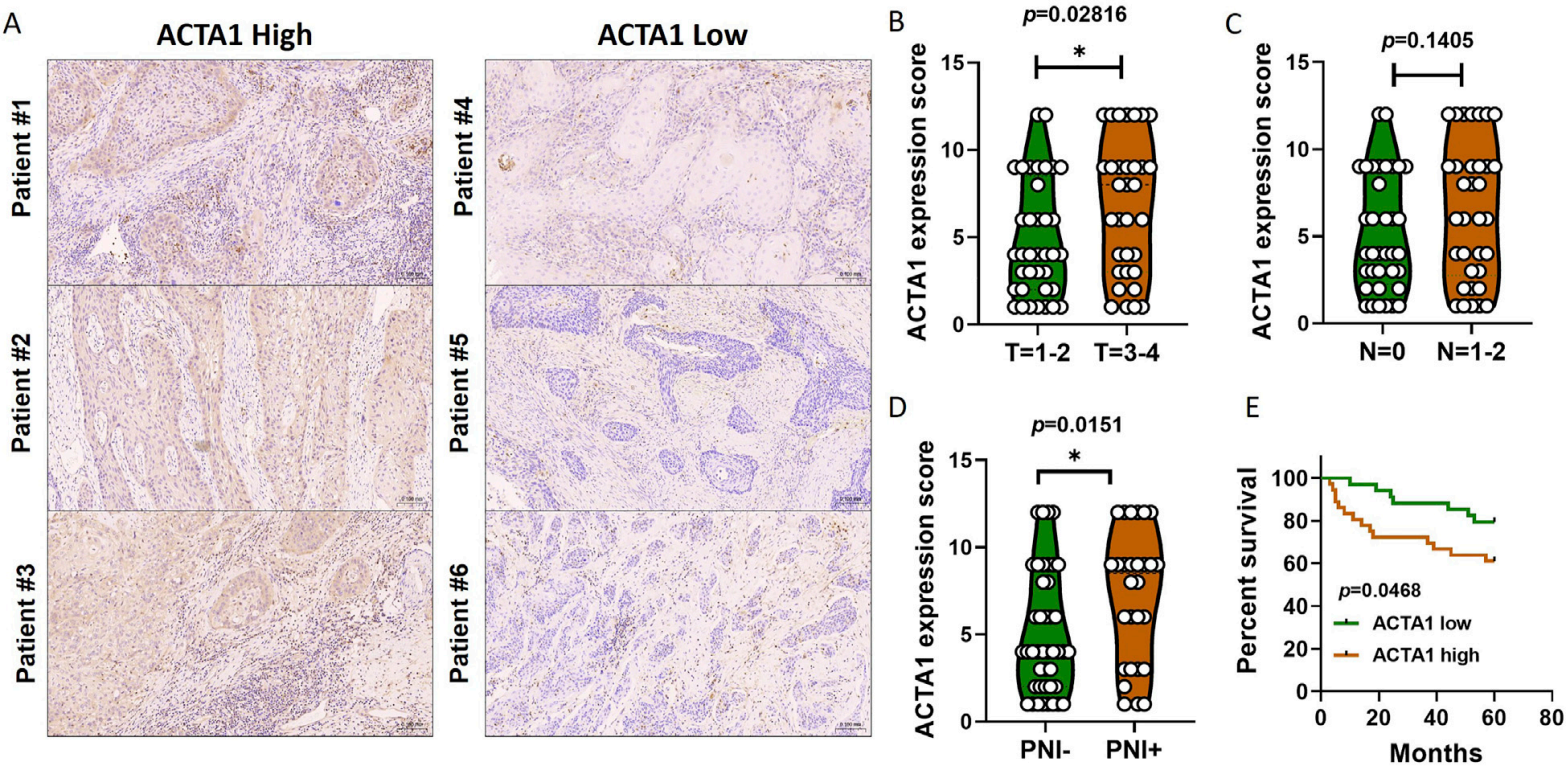
Immunohistochemistry was used to detect the coincidence degree of ACTA1 expression with tumor cells in OSCC serial sections **(A)**. The expression of ACTA1 was higher in the tumors of patients with T3 and T4 stages (*p =* 0.02816) **(B)**. There was no statistically significant difference in lymph node metastasis (*p =* 0.1405) **(C)**. The expression of ACTA1 in the tumor cells of the PNI^+^ group was significantly higher than that of the PNI^−^ group (*p =* 0.0151) **(D)**. The group with high ACTA1 expression had a worse prognosis (*p =* 0.0468) **(E)**.

In this study, a total of 70 tumor tissues from patients with oral squamous carcinoma were collected (Table 2). Immunohistochemical staining of the actin α1 was performed (Figure 6A) and the expression was scored. The results showed that the expression of actin α1 was higher in the tumors of patients with T3 and T4 stages (Figure 6B), and although there was no statistically significant difference in lymph node metastasis, there was a trend of higher actin α1 scores in patients with lymph node metastasis (Figure 6C), and the expression of actin α1 in the tumor cells of the PNI^+^ group was significantly higher than that of the PNI^−^ group (Figure 6D). The patients were divided into two groups according to actin α1 immunohistochemistry scores, and the survival analysis showed that the group with high actin α1 expression had a worse prognosis (Figure 6E).

**FIGURE 7:**
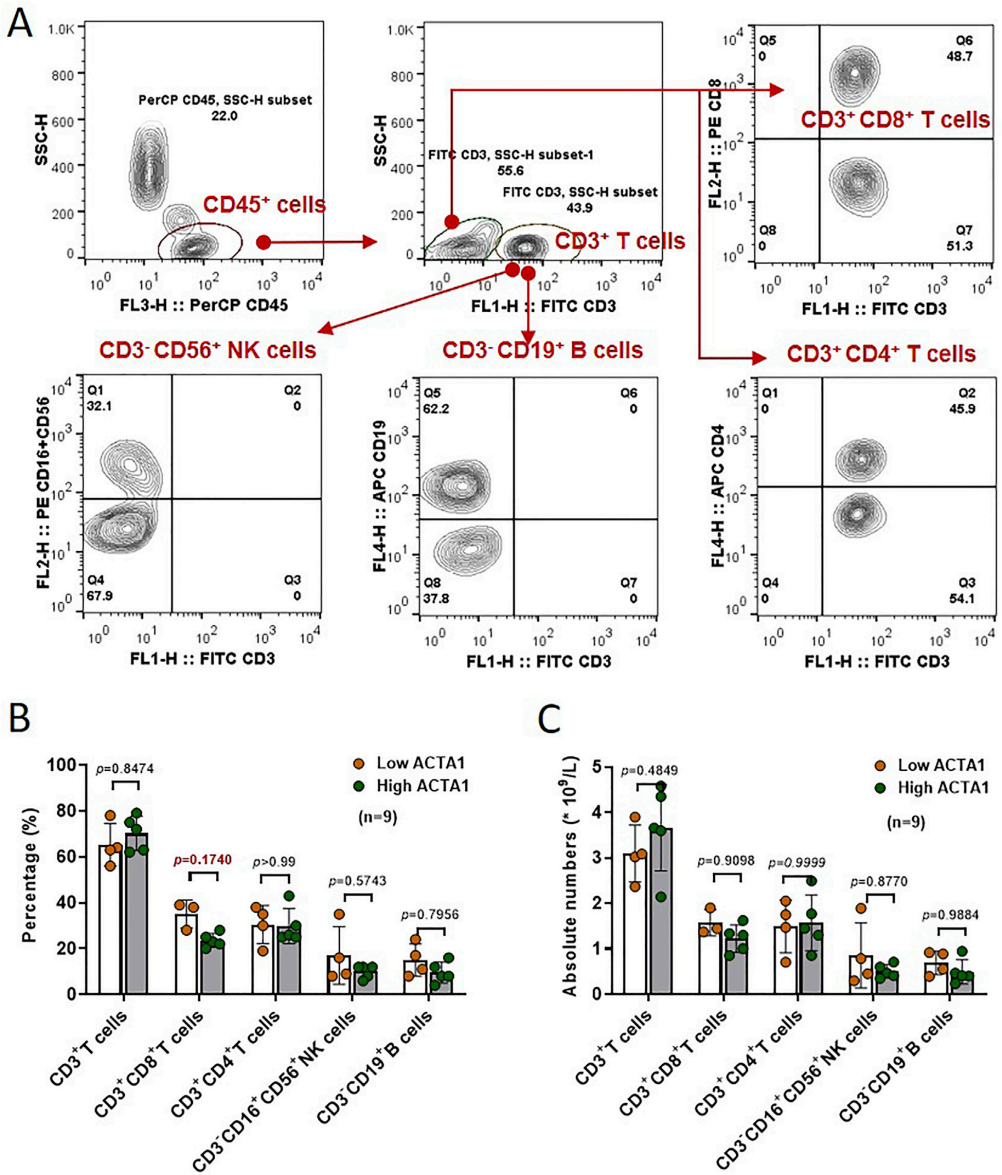
The ratio and absolute number of human CD3^+^ T cells, CD3^+^CD4^+^ helper/inducer T cells, CD3^+^CD8^+^ cytotoxic T cells, CD3^−^CD19^+^ B cells, and CD3^−^ CD16^+^ and/or CD56^+^ NK cells **(A)** in peripheral blood were analyzed in High-ACTA1 and Low-ACTA1 groups using the BD Multitest™ reagent **(B, C)** (n = 9 patients were missed), by multiple t-test.

We next analyzed the ratio and absolute number of key immunocytes in peripheral blood of OSCC patients (n = 9) according to actin α1 expression by flow cytometry. Human CD3^+^ T cells, CD3^+^CD4^+^ helper/inducer T cells, CD3^+^CD8^+^ cytotoxic T cells, CD3^−^CD19^+^ B cells, and CD3^−^CD16^+^, and/or CD56^+^NK cells were analyzed in actin α1^high^ and actin α1^low^ groups, and the strategy for gating lymphocytes is shown in Figure 7A. Although there were no statistically significant differences in the comparisons due to the small number of samples, the results indicated that patients with enhanced actin α1^+^tumor cells had relatively low ratio of CD3^+^CD8^+^ cytotoxic T cells (Figures 7B, C).

## Discussion

In this study, by analyzing the clinical case characteristics and survival prognosis of 279 OSCC samples within the TCGA database grouped according to whether or not PNI occurred, we found that PNI was detected more frequently in tumors of patients at T3 and T4 stages or Stage III and Stage IV, indicating that tumor cell invasion of the tumor microenvironment within the tumor microenvironment is more likely to occur when oral cancer tumors develop into intermediate or advanced stages of the neural tissue. With the increased study of neural tissue in cancer microenvironment, new theories of nerve-cancer crosstalk have now been proposed (Silverman et al., 2021). Whether this phenomenon occurs as an inevitable phenomenon that occurs passively as the tumor grows, invades more normal tissues, and potentially comes into contact with more nerve tissues, or whether it is the result of active invasion of nerve tissues by tumor cells with higher invasive ability, has not been reported in the literature, and further research is underway in this study. In the survival prognostic analysis, we found that the overall survival time was significantly shorter in samples that developed PNI compared with those that did not, a result that is consistent with the conclusion of most studies that PNI is one of the poor prognostic factors for oral squamous carcinoma. This may be due to the fact that nerve infiltration makes it easier for tumor cells to migrate and spread along the nerve bundles, increasing the risk of distant metastasis (Wan et al., 2021; Xiong et al., 2023; Weitz et al., 2023). Also, nerve infiltration may affect the tumor’s response to treatment, making it less effective.

The results of the two groups of samples in terms of immune score, stroma score, and ESTIMATE Score showed significant differences between the two groups in terms of stroma score, whereas the differences in terms of immune score and ESTIMATE score were not significant. This result may imply that nerve infiltration mainly affects the interaction between the tumor and the stroma, while the effect on the immune system may be relatively minor. However, this still needs to be confirmed by further studies.

In addition, we mapped the dataset of OSCC samples to inhibitor-treated melanoma samples using the SubMap algorithm and preliminarily predicted the differences in PNI subgroups on immunotherapy. The results showed that nerve-infiltrating samples were more sensitive to CTLA4 inhibitors, while sensitivity to PD-1 inhibitors did not reach a significant level. And the proportion of T cells follicular helper was significantly higher in the PNI^−^ samples than in the PNI^+^ samples, and only macrophages, especially the M2 tumor-associated macrophages was significantly higher in the PNI^+^ OSCC. CTLA-4 is one of the important targets for tumour immunotherapy. By blocking the CTLA-4 signalling pathway, the inhibitory state of T cells can be lifted and the anti-tumour immune response can be enhanced (Vetizou et al., 2015). For example, Yervoy (Ipilimumab) manufactured by Bristol-Myers Squibb is the world’s first approved and marketed CTLA-4 antibody drug, which has been used for the treatment of a variety of cancers (Rohaan et al., 2022). Combining these 2 results, we can speculate that CTLA-4 can upregulate T cells in PNI^+^OSCC tumours and thus have an immunotherapeutic effect. This finding provides clues for developing personalized immunotherapy strategies for patients with different subtypes of oral squamous carcinoma. However, it is important to note that these predictive results are only preliminary explorations and further clinical trials are needed to validate their effectiveness.

We extracted the differentially expressed genes and analyzed the functional enrichment of the differentially expressed genes between the PNI^+^ samples and the PNI^−^ samples using the limma package. The results showed that there were significant gene expression differences between the two groups of samples, and that these differential genes were mainly involved in aspects of muscle structure and movement. We selected the ACTA1 gene, which was significantly highly expressed in the PNI-positive group, for validation. Actin is a key structural protein that constitutes the cytoskeleton and plays a role in functions such as cell division, migration and vesicular transport (Dominguez and Holmes, 2011). It consists of six cell type-specific isoforms: ACTA1, ACTA2, ACTB, ACTC1, ACTG1, and ACTG2. Abnormal expression of actin isoforms has been reported in a number of cancers. ACTA1 is a gene encoding actin α1, which is the major isoform found in skeletal muscle and is essential for muscle contraction (Wang et al., 2022). High expression of ACTA1 has been reported to be associated with shortened survival in oral squamous cell carcinoma (Dai et al., 2020). In addition, in basal-like breast cancer, ACTA1 is a biomarker associated with chemotherapy resistance. There are many possible mechanisms by which ACTC1 protein promotes tumorigenesis. One may be through annexin, which is a Ca2^+^-dependent phospholipid-binding protein that plays a role in vesicle trafficking, cell proliferation, and apoptosis (Iaccarino et al., 2011). Annexin expression has been associated with shortened survival, tumorigenesis, and progression of malignant ovarian cancer (Li et al., 2022). Notably, annexin physically interacts with ACTA1 (Saxena et al., 2012). In pancreatic ductal adenocarcinoma, ACTA1 expression is a feature of cancer-associated fibroblasts (Tsang et al., 2013). Stromal progenitor cells and fibroblast-like cells show increased ACTA1 expression when they become cancerous (Barisic et al., 2020). Coincidentally this change in cell morphology also occurs during the epithelial-mesenchymal transition when cells undergo malignant transformation and confers a more migratory phenotype. Immunohistochemical experiments verified that high expression of ACTA1 was observed in tumor tissue samples undergoing PNI, and also the prognosis was worse in the ACTA1^high^ group, which was largely consistent with previous bioinformatics predictions and reports by other scholars. Although there was no statistically significant difference in T stage and LNM between the 2 groups of patients, there was still a tendency for a higher proportion of T stage 3-4, and the reason for the lack of a statistically significant difference may be related to the sample size.

Based on the function of actin α1 protein, we hypothesized that after tumor cells invaded nerve tissues, the invaded nerves could upregulate the ACTA1 expression of tumor cells, which led to epithelial-mesenchymal transition of the cells, promoting invasion and metastasis of the tumor cells, resulting in a worse prognosis. However, the specific regulatory mechanism may be related to the neurotransmitters secreted by nerves, ACTA1 protein affecting cytoskeletal structure, intercellular junctions, or extracellular matrix degradation, which has not yet been reported in detail and still needs to be further explored.

In summary, this study revealed the clinical features and prognostic significance of nerve infiltration in oral squamous carcinoma by analyzing OSCC samples within the TCGA database, and initially explored its relationship with immunotherapy sensitivity and gene expression. What’s more, PNI^+^ OSCC patients with upregulated of Actin α1 could benefit from cytotoxic T cell-mediated immunotherapy. However, these results are only preliminary explorations, and further studies are needed to validate and deepen our understanding of nerve infiltration in oral squamous carcinoma.

## Data Availability

The original contributions presented in the study are included in the article/supplementary material, further inquiries can be directed to the corresponding author.
